# COP26 and Health: Some Progress, but Too Slow and Not Enough

**DOI:** 10.5152/TJAR.2022.08022022

**Published:** 2022-02-01

**Authors:** Laurie Laybourn-Langton, Richard Smith

**Affiliations:** 1 Senior Adviser, UK Health Alliance on Climate Change; 2 Chair, UK Health Alliance on Climate Change

*The health community must step up its efforts to hold countries accountable for reducing greenhouse emissions and promoting adaptation*

The editorial on climate change and biodiversity published in over 220 health journals in September had two main demands: keep global temperature increases below 1.5°C above pre-industrial levels to avoid catastrophic damage to health; and accept that this can be achieved only by rich countries making bigger cuts in greenhouse gas emissions and transferring substantial resources to the countries most vulnerable the effects of climate change.^1^ Neither demand was fully met at COP26 in Glasgow. The editorial was also aiming to make the voice of the health community more prominent in global discussions on climate change and environmental destruction. Some progress was made with this aim, but again not enough.

Although the mantra of COP26 was “keep 1.5°C alive,” the pledges made by countries to reduce emissions are insufficient to keep the temperature rise to below 1.5°C. Before COP26, the United Nations estimated that current pledges will lead to an increase of 2.7°C, a level that would lead to devastating effects on health through extreme weather events, crop failure, water shortages, forced migration, conflict, and a rise in sea level that will mean the disappearance of some island countries.^2^ Even with the additional pledges made at COP26, temperatures are expected to rise well above 2°C.^3^

Christina Figueres, the head of the UN climate change convention in 2015 that achieved the Paris agreement, argues, however, that COP26 has made the aim of 1.5°C widely accepted, removing the aim of “below 2°C” that emerged in Paris.^4^ Countries are now required to review their pledges—called Nationally Declared Contributions (NDCs) in UN speak—every year rather than every five years as at present. There is, however, no system of enforcement, and countries often fail to meet the pledges they make. Promises are easy; implementation is hard.

For the first time the final COP26 agreement mentioned fossil fuels, the source of most of the greenhouse gases.^5^ Countries agreed to accelerate “efforts towards the phasedown of unabated coal power and phase-out of inefficient fossil fuel subsidies.” Countries like India and China that depend heavily on coal for their energy supply insisted on the word “phasedown” of coal rather than the original “phase out.”^6^ It is a small success to have coal and fossil fuels mentioned in the final agreement, but at the same time the weak wording is a sign of the absolute failure of the world to adequately address the crisis. 

The $100bn support for low income and other vulnerable countries, which was promised back in Paris, did not materialise in Glasgow. It is now expected by 2023, deepening antagonisms between rich and vulnerable countries over the inequity of the global response to phasing out fossil fuels. There was, however, a greater emphasis on the need for more adaptation funding, as the editorial in the journals requested. Countries and their people are recognising that climate change is here now not in the future. Vulnerable countries wanted a "Glasgow loss and damage facility," which would see funds passing from rich countries to vulnerable countries as compensation for the damage the rich countries have caused and continue to cause. Rich countries squashed this facility, greatly angering the vulnerable countries. 

The editorial in the health journals sought to connect the climate element of the environmental crisis with other damage to nature, including biodiversity loss, deforestation, harm to the oceans, and soil destruction. COP26 did see $20bn committed for forest protection, and more than 100 countries, including those with the largest forests, pledged to reverse deforestation by 2030 at the latest – though a similar pledge had already been made in 2014. Generally, however, broader damage to nature did not feature, which is partly because the UN process largely creates a separation between climate change, the focus of COP26, and biodiversity, which is being considered next year at a conference in China.

Business featured prominently at COP26. If the world is to reach net-zero then business—like every other activity—will have to play its part. Many businesses have committed to reach net-zero and, perhaps more importantly, investors have discovered that there is money to be made from investing in genuinely green projects and money to be lost by investing in fossil fuels, which are rapidly becoming stranded assets. However, net zero pledges made by businesses have attracted considerable doubts – and many remain full of loop holes, including allowing for continued investment in fossil fuels - leading the climate activist Greta Thunberg to call the conference “a global north greenwash festival, a two-week long celebration of business as usual and blah blah blah.”^7^ In response, the UN Secretary General has committed to establishing a “greenwashing” watchdog.^8^

Health was more prominent in COP26 than in any previous COPs in that the WHO had a health pavilion for the first time and health had an hour-long session with ministers in the main part of the meeting. The health pavilion featured dozens of sessions, most of which are available online.

Patricia Espinosa, the executive secretary of the United Nations Framework Convention on Climate Change, was expected to appear alongside the UK’s senior health minister at the health session in the main part of the meeting, but neither attended. The meeting did, however, feature two British ministers, representatives of Fiji and Egypt governments, a former British prime minister, a senior official from the US government, the chief executive of GSK, and others. The representative from Fiji said that in his region more people are already dying from climate change that any other cause, and the US representative told the audience that the US accounts for a quarter of all global emissions from health systems, which if they were a country would be the fifth largest emitter of greenhouse gases. Most health systems currently have rising emissions.^9^

Fifty countries committed at COP26 to “take concrete steps towards creating climate-resilient health systems.”^10^ Argentina, Fiji, Malawi, Spain, the United Arab Emirates, the US, and 39 others will achieve low-carbon, sustainable health systems, while Bangladesh, Ethiopia, the Maldives, the Netherlands, and 45 others have committed to enhance the climate resilience of their health systems.

Nobody knows how to achieve net zero within a health system, but we do know that everything, including clinical practice, will have to change; about two thirds of the emissions come from suppliers, meaning that they too will have to reach net zero; and research and innovation will be essential. Funding for research on climate change and health has been small, but the UK minister announced a new fund for research on climate change and health.

Despite greater attention to health, the word health appeared only once in the final document agreed at the meeting: "[countries,] when taking action to address climate change, respect, promote and consider their respective obligations on…the right to health.” ^5^

John Kerry, the US climate envoy who was at the original earth summit in Rio de Janeiro in 1992 and deeply involved in negotiating the agreement at the Paris COP, acknowledged that COP26 was never going to solve the climate crisis completely. But, he said, “Paris built the arena, Glasgow starts the race…When we leave Glasgow, our password will be implementation, follow-up and follow-up.”^11^

His words ring true for the health community. Restricting the rise in global temperature to 1.5°C is still possible with emergency action, and we must continue to emphasise the extreme danger to health from temperatures rising above 1.5°C and the great benefits to health that can result from countries decarbonising their economies. We must encourage countries to be bolder in cutting emissions, promoting adaptation, supporting vulnerable countries – and do more to hold them to account. We must also concentrate on implementation, particularly within health systems where we have most influence. 
